# The Journey of Business Opportunity Evaluation: When and Why Does Opportunity Novelty Promote Vs. Inhibit Opportunity Adoption?

**DOI:** 10.3389/fpsyg.2021.732565

**Published:** 2021-10-12

**Authors:** Lin Lin, Yanan Lin, Song Lin

**Affiliations:** Business School, Central University of Finance and Economics, Beijing, China

**Keywords:** cognitive evaluation, opportunity novelty, adoption, construal level, creativity perception, risk perception

## Abstract

As a typical characteristic of entrepreneurial opportunities, novelty is essential for firms to establish and maintain a sustainable competitive advantage under the current complex and dynamic business environment. However, why is it that some entrepreneurs adopt novel opportunities but others do not. Little is known about the precise nature of cognitive evaluation for opportunity novelty. Drawing upon information processing theory and construal level theory (CLT), we propose that the effects of opportunity novelty on adoption decisions depend on entrepreneurs' construal level through which information is processed. We design an experiment and find partial support for our hypotheses. Results indicate that entrepreneurs using a low-level construal perceive more risk for opportunity novelty, which in turn decreases the possibility of opportunity adoption. Meanwhile, opportunity novelty also positively influences entrepreneurs' creativity perception, which in turn increases the possibility of opportunity adoption. But construal level does not play any role in this evaluation path. Taken together, the findings improve our understanding of “how entrepreneurs evaluate an opportunity based on its objective characteristics” by providing empirical insights into the cognitive information processing process from opportunity novelty to adoption. Additionally, we discuss implications for entrepreneurial practice and future research.

## Introduction

Novelty is always viewed as a typical characteristic of entrepreneurial opportunities (Wood and Williams, 2014; Hilmersson et al., 2021) as entrepreneurship is a process associated with novelty and value creation in economic activities (Davidsson and Wiklund, 2001). *Opportunity novelty* refers the originality, newness, and distinctiveness of opportunities compared with existing products or services in the market (Perry-Smith and Mannucci, 2017). Some scholars argue that entrepreneurship opportunities are essentially a continuum between two extreme types of replicated opportunities and innovative opportunities, and the position of a particular one in which depends on its novelty compared to the existing means and/or ends relationship (Sarasvathy et al., 2003). In other words, all opportunities have varying degrees of novelty, ranging from multiple stages of incremental to radical innovation (Criscuolo et al., 2017). Prior research has shown that opportunity novelty is essential for firms to establish and maintain a sustainable competitive advantage under the current complex and dynamic business environment (MacDonald and Ryall, 2004; Knudsen and Levinthal, 2007), which is often deemed as a proxy for the value creating potential of opportunities in the future (Hilmersson et al., 2021). However, we often see frequently that firms make different decisions for novel opportunities in commercial practices. For example, Apple, a world-famous high-tech company, adopted the novel opportunity and became one leader in the global smartphone market. By contrast, Kodak, a firm that invented the first digital camera, gave up a novel opportunity to fully enter the digital industry. Why is it that some entrepreneurs adopt novel opportunities but others do not? In fact, the evaluation process from opportunity novelty to opportunity adoption decisions that refer to the result of entrepreneurs rely on different criteria in deciding whether to adopt an opportunity for further development and implementation (Škerlavaj et al., 2014; Chan et al., 2018) remains largely unanswered in entrepreneurship but are central to understanding the process of business creation (Haynie et al., 2009).

Opportunity evaluation, an essential inflection point (Scheaf et al., 2019) is crucial for entrepreneurs to determine whether to further develop and exploit opportunities, switch to alternative opportunities, or give up entrepreneurial actions completely (Wood and McKinley, 2010; Gruber et al., 2015). Theoretically, the evaluation process from opportunity novelty to adoption is an integral part of opportunity evaluation. However, entrepreneurial scholars are more concerned with opportunity recognition and exploitation compared with opportunity evaluation (Wood and McKelvie, 2015). Meanwhile, the existing literature mentioning opportunity evaluation mainly emphasizes the effects of individual characteristics and their cognitive differences, such as gender (Gupta et al., 2013), role identities (Mathias and Williams, 2015), emotions (Welpe et al., 2012; Zhao and Xie, 2020), risk propensity (Keh et al., 2002), prototypes (Baron and Ensley, 2006), and analogy explanation (Uygur, 2017) as well as structural alignment (Grégoire and Shepherd, 2012), which has formed a growing research stream (Nicolau et al., 2009). The issue of how objective characteristics of opportunities influence individuals' opportunity adoption decisions is rarely discussed. In addition, novel opportunities usually lack sufficient and specific information because of unfamiliarity (Chan et al., 2018), which challenges the cognitive abilities of entrepreneurs (Ocasio, 1997) and increases the difficulty of adopting the most promising and favorable opportunity. It is unclear that which cognitive processes are available for entrepreneurs when they need to make adoption decisions for novel opportunities. Therefore, we aimed to explore the following question: ***How do entrepreneurs evaluate opportunity novelty to make adoption*** ***decisions?***

Previous research has shown that differences in how decision-makers process information are affected by their interpretation of events, which in turn acts on their ultimate choices (Walsh, 1995). Construal level, the extent of people representing events from concrete to abstract (Trope and Liberman, 2003, 2010), plays an important role in interpreting objective information (Mount et al., 2021). Construal level theory (CLT) emphasizes the importance of an individual's understanding of the environment (Trope and Liberman, 2010). According to CLT, opportunity novelty can be interpreted differently depending on whether an entrepreneur uses a high-level or low-level construal, which may strengthen or weaken individuals' perceptions and judgments. Usually, individuals with high-level construals will form relatively abstract, coherent, and superordinate mental representations of events, focusing on why one performs them (Trope and Liberman, 2003, 2010). In contrast, individuals with low-level construal will form relatively concrete and contextualized mental representations of events, focusing on how one performs them (Trope and Liberman, 2003, 2010). However, literature on the effect of construal level on the evaluation process is still scarce. Therefore, we included construal levels into our study and explore ***whether the cognitive evaluation process, from opportunity*** ***novelty to adoption decision-making, would be shaped by an individual's construal*** ***level*.**

Against this backdrop, our paper, based on information processing theory and CLT, explores the cognitive evaluation process from opportunity novelty to opportunity adoption across an experimental method. We propose that opportunity novelty and construal level interact to influence opportunity adoption by changing entrepreneurs' perceptions. Specifically, *creativity perception* refers to the extent to which an opportunity is perceived subjectively to be novel and useful (Mount et al., 2021). And *risk perception* is defined as the subjective judgment of the amount of risk inherent in the situation (Allinson et al., 2000; Keh et al., 2002). According to CLT, we predict that creativity perception will be strengthened but risk perception will be weakened when an entrepreneur interprets a novel opportunity with a high-level construal, which emphasizes its superordinate and decontextualized features (Trope and Liberman, 2003, 2010) thereby stressing creativity perception and deemphasizing risk perception. On the contrary, creativity perception will be weakened but risk perception will be strengthened when an entrepreneur interprets a novel opportunity with a low-level construal, which emphasizes its subordinate and contextualized features (Trope and Liberman, 2003, 2010) thereby stressing risk perception and deemphasizing creativity perception. Ultimately, these processes will, in turn, influence opportunity adoption differently.

Our study makes several contributions. First, this paper, drawing upon information processing theory and CLT, adds to the literature on opportunity evaluation and improves our understanding of “how entrepreneurs evaluate an opportunity based on its objective characteristics” by providing empirical insights into the cognitive evaluation process from opportunity novelty to opportunity adoption, which is often ignored by entrepreneurial scholars (Haynie et al., 2009). We demonstrate that the novelty of opportunity is closely related to the subjective perception of entrepreneurs in terms of gains and losses, which, in turn, is associated with decisions of opportunity adoption. Second, our research contributes to the literature on CLT more broadly by introducing construal level to the process of entrepreneurship opportunity evaluation and positioning it as an essential cognitive variable, which provides us a better understanding of the fact that entrepreneurs' construal level interacts with opportunity novelty to shape their subjective perceptions and ultimately influence their decisions of opportunity adoption. Opportunity evaluation is a cognitive phenomenon (Krueger, 2000). The way how entrepreneurs process and interpret information, as everyone knows, plays a vital role in their responses to opportunities (Mount et al., 2021). Thus, this paper provides new empirical support for the role of construal level in entrepreneurial decisions and further responds to the call of unpacking the cognitive foundations of entrepreneurs' information processing by some scholars (Steinbach et al., 2019). Additionally, there are several implications for entrepreneurial practice and education as it related to intervention techniques to promote adoption decisions for novel opportunities from a cognitive perspective.

## Theory and Hypotheses Development

### Information Processing Theory

Cognition is one of the significant approaches to distinguish entrepreneurs from other groups of people and explain how individuals make decisions (Das and Teng, 1997), which focuses on their preferred ways of collecting, processing, and evaluating information (Allinson et al., 2000). Indeed, opportunity evaluation is essentially an intensive cognitive process (Shepherd et al., 2007; Mount et al., 2021). Whether entrepreneurs represent external stimuli as actionable signals is related to the cognitive judgment generated by the process of opportunity evaluation (Autio et al., 2013). Such a cognitive phenomenon can help understand better why some people adopt and exploit opportunities while others do not (Shane and Venkataraman, 2000).

According to information processing theory, the cognitive process of an individual is essentially the process of information processing, which can be portrayed as a dynamic process in which information (what people see, hear, read, and think) is processed in multiple stages in the human mind (Ingram, 1984). Meanwhile, the process of objective information from perceived by individuals to transformed into their behaviors will be influenced by many factors. Personal cognitive factors can affect how perceivers process information (Zhou et al., 2017). Individuals' construal level, purely cognitive orientation (Fujita and Sasota, 2011), is one critical factor that can shape the evaluation process (Liberman and Förster, 2009; Mount et al., 2021). Construal level theory believes that people will cognitively represent (“construe”) objects at different levels ranging from concrete to abstract levels (Liberman and Trope, 2008; Trope and Liberman, 2010). The difference between concrete and abstract construals of objects has been of central importance in person perception research (Trope, 1986; Trope and Liberman, 2003). Individuals draw different perceptions and inferences for the same information due to multi-level construals (Trope and Liberman, 2003), which in turn affects their final decisions (Förster et al., 2004). Therefore, we suggest that interacting with opportunity novelty, construal level will join in shaping the opportunity evaluation process.

The human information processing model, as the most classic theoretical model of information processing theory, believes that individuals' cognition of objects and events originates from their perceptions and is ultimately output as behavioral responses (Atkinson and Shiffrin, 1968). In other words, individuals make decisions based on the perceptions of one target instead of its characteristics (Podoynitsyna et al., 2012). As such, drawing upon information processing theory, we propose that the evaluation of opportunity novelty is an information processing process during which entrepreneurs gain perceptions about novelty with different construal levels and then decide whether to adopt opportunities. However, under a rapidly changing and complex business environment with a large number of external stimuli, entrepreneurs often face tremendous challenges to filter out the most favorable novel opportunities (Chan et al., 2018). Thus, they may adopt shallow processing methods with simplified decision clues to search for the most valuable opportunities in the process of information processing due to the limited ability to process all stimuli in the environment (Piezunka and Dahlander, 2015).

Scholars assert that a novel opportunity may be associated with different perception concepts (Zhou et al., 2017). It may be associated with positive concepts such as “gain” or negative concepts such as “loss” (Gawronski and Bodenhausen, 2006). For example, entrepreneurs, depending on opportunity-specific attributes (Wood and Williams, 2014), pay more attention to potential return (Ardichvili et al., 2003), and loss (Keh et al., 2002; Foo, 2011). It means the content of entrepreneurs' perceptions for a novel opportunity encompasses both gain- and loss-side issues during they evaluate the worth of the opportunity and make final judgments. Accordingly, we identified creativity perception representing “gain-side” aspects and risk perception representing “loss-side” aspects in our study as key heuristic cues that guide entrepreneurs' opportunity adoption decisions, which can simplify the process of searching for the most favorable opportunity clues in a large amount of complex novel information with the least effort.

Specifically, entrepreneurship is essentially an act of creativity as the key to entrepreneurial opportunities involves introducing new means-ends relationships such as the creation of new products, new business models, or new technologies (Shane and Venkataraman, 2000). Thus, opportunity evaluation is future-focused, such that entrepreneurs have to evaluate potential competitive advantage and the firm's gains for the undeveloped opportunity (Haynie et al., 2009). As the engine of future business growth, creativity drives differentiation, and competitiveness (Zhou et al., 2017). Creativity implies distinctiveness and rarity (Porter, 1980), which can increase the possibility of future benefits (Choi and Shepherd, 2004). Generally, the more creative the opportunity, the greater the potential return (Wood and Williams, 2014). In a word, creativity, implying the potential value of the opportunity, provides significant gain-side advantages. Thus, we suggest that creativity perception is a vital heuristic cue representing “gain-side” aspects and the critical evaluation criterion when entrepreneurs evaluate opportunity novelty.

**Figure 1:**
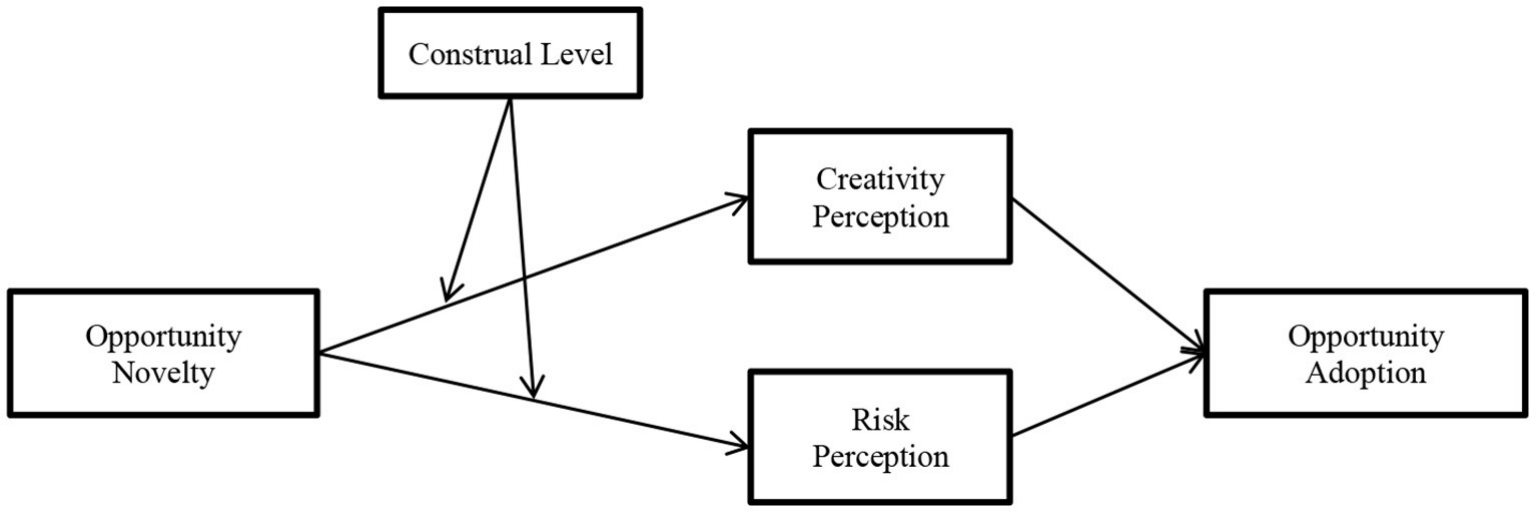
The conceptual framework.

Meanwhile, risk is another critical element in various decision-making contexts for entrepreneurs (Forlani and Mullins, 2000). Consistent with previous literature, risk perception is a significant “loss-side” aspect of how entrepreneurs evaluate available opportunities (e.g., Shane and Venkataraman, 2000; Kim et al., 2010; Wood and Williams, 2014). Opportunity evaluation is future-oriented (Haynie et al., 2009), which causes entrepreneurs to make judgments and decisions under uncertain conditions (Allinson et al., 2000; Uygur, 2017). Uncertainty is usually accompanied by risk (Lin et al., 2019), and risk refers to the possibility that entrepreneurs successfully turn novel opportunities into new products/new services (Keh et al., 2002). Risk means the possibility of loss. Entrepreneurs who fail to start a business venture may suffer huge losses (Scheaf et al., 2019). Empirical evidence has demonstrated that risk perception plays an essential role in the decision to adopt an opportunity and start a venture (Keh et al., 2002). As such, we suggest that risk perception is a salient evaluative criterion of “loss-side” aspects that naturally comes to entrepreneurs' minds when they evaluate the novelty of opportunities. In summary, we presented our conceptual framework in Figure 1.

### The Interactive Effect of Opportunity Novelty and Entrepreneurs' Construal Level

Perceptions of creativity or risk are likely to be influenced by the interaction of objective opportunity novelty and subjective construal level as individuals can interpret the same object differently depending on a high-level or low-level construal (Trope and Liberman, 2010). High-level construals are associated with abstract information processing, whereas low-level construals are associated with concrete information processing (Trope and Liberman, 2010). Drawing on CLT, we argue that entrepreneurs will perceive more creativity and less risk in novel opportunities when they use a high-level construal compared with a low-level construal.

Entrepreneurs using high-level construals are future-oriented and pay more attention to abstract and broad information related to the valence of distal goals and profits (Trope and Liberman, 2003). The greater they are psychologically distant from an opportunity, the more likely they are to emphasize its creativity (Förster, 2009). Thus, entrepreneurs will perceive higher creativity that predicts future returns for a novel opportunity with a high-level construal. Meanwhile, entrepreneurs using a high-level construal may be less likely to experience uncertainty about novel opportunities as their mindsets fit the content under consideration (Mueller et al., 2014). Thus, they will perceive less risk. As the evidence showed by Berg (2016) in his study, it is opposite between creativity perception and risk perception for individuals when they evaluate a novel opportunity with the same level of construal.

Entrepreneurs using low-level construals, in contrast, are present-oriented and tend to focus on concrete and narrow information related to specific methods on how to achieve goals (Wiesenfeld et al., 2017). They pay more attention to current losses compared with future gains. New products or services generated from novel opportunities are often associated with lower market legitimacy and higher uncertainty because they deviate from existing products or services in the market (DiMaggio and Powell, 1983; Giachetti et al., 2017). Thus, entrepreneurs may perceive more uncertainty for novel opportunities (Mount et al., 2021), which in turn leads to more risk perception. Meanwhile, given that the more concrete processing orientation presents a mismatch with opportunity novelty, a low-level construal may lead to less creativity perception (Mueller et al., 2014). Indeed, prior research indicated that using a low-level construal to interpret novel events decreases individuals' creativity perception (Förster et al., 2004).

Thus, we predict the following:

*Hypothesis 1: Opportunity novelty and construal level will interact to influence creativity perception such that the relationship will be more positive when construal level is high as compared to when construal level is low*.
*Hypothesis 2: Opportunity novelty and construal level will interact to influence risk perception such that the relationship will be more positive when construal level is low as compared to when construal level is high*.

### The Mediating Effect of Creativity Perception and Risk Perception

We further argued that entrepreneurs who perceive heightened creativity perception resulting from the interaction between opportunity novelty and high-level construal will be more likely to make decisions to adopt opportunities. In contrast, entrepreneurs who perceive heightened risk perception resulting from the interaction between opportunity novelty and low-level construal will be less likely to make decisions to adopt opportunities.

Literature on the resource-based view suggests that firms should choose valuable, rare, and inimitable resources to establish competitive advantages (Barney, 1991; Helfat and Peteraf, 2003). Creativity composed of novelty and usefulness (Mueller et al., 2018) usually means differentiation and potential profits (Woodman, 2008). Specifically, something new cannot be easily imitated and provides an entrepreneur with differentiation to generate and maintain competitive advantages (Rumelt, 1991). The novelty of creativity may also bring first-mover advantages (Liberman and Montgomery, 1988). Meanwhile, the usefulness of creativity guarantees that customer needs are better served and allows entrepreneurs to obtain potential economic benefits (Gruber et al., 2015). As such, entrepreneurs will be more optimistic about entrepreneurial success in the future and more likely to adopt novel opportunities when they perceive higher creativity for opportunity novelty with a high-level construal.

On the contrary, empirical evidence demonstrated that risk perception affects entrepreneurs' decisions about opportunity evaluation as it is associated with losses (Keh et al., 2002). Opportunity exploitation usually requires a large number of resources in terms of time, money, effort as well as social capital (Haynie et al., 2009). Once failed, entrepreneurs will suffer great losses in addition to potential returns (Foo, 2011; Scheaf et al., 2019). Fundamentally, risk perception captures the possibility of failure (Scheaf et al., 2019). As such, entrepreneurs will be less likely to adopt opportunities when they perceive higher risk for opportunity novelty with low-level construals.

In sum, combining these arguments with the proposed interactive effect of opportunity novelty and construal level on creativity perception (*H1*) as well as risk perception (*H2*), we predict the following:

*Hypothesis 3: Creativity perception will mediate the interactive effect of opportunity novelty and construal level of entrepreneurs on opportunity adoption such that the indirect effect is more positively significant when construal level is high as compared to when construal level is low*.
*Hypothesis 4: Risk perception will mediate the interactive effect of opportunity novelty and construal level of entrepreneurs on opportunity adoption such that the indirect effect is more negatively significant when construal level is low as compared to when construal level is high*.

## Methods

### Participants and Procedure

To improve causal inferences, we designed an experiment with college student participants to test hypotheses 1, 2, 3, and 4. Following the common practice in prior literature of a similar nature, we use college students as our samples (e.g., Welpe et al., 2012; Gupta et al., 2013; Uygur, 2017). Several reasons why we choose student samples are as follows. First of all, compared with real entrepreneurs, it is convenient to access student samples, which ensures a sufficient sample size. Ideally, data for entrepreneurial research should be obtained from entrepreneurs or entrepreneurial teams (Kim et al., 2010). However, entrepreneurs or top-level executives in new ventures are usually less willing to respond to research invitations as they tend to work in a very time-restricted and high-stress environment (Cooper and Baglioni, 1988; Grichnik et al., 2010), so that it is tough to study such samples. Most importantly, previous research has shown that individual cognitive evaluation is fundamental by nature (Welpe et al., 2012). There are no differences between a student sample and a sample of entrepreneurs or the general population (Uygur, 2017). Our study aims to examine individuals' decision processes of opportunity adoption before starting ventures when they face novel opportunities. In other words, we focus on, rather than established entrepreneurs and entrepreneurial experience, the question of why and how people make judgments about whether to adopt novel opportunities or not. Therefore, for the antecedents of entrepreneurship, students are a good sample because individuals with higher education are more likely to become entrepreneurs (Shane and Venkataraman, 2000).

As such, we recruited 80 Chinese college students (55% female; *M*_age_ = 22.19, *SD* = 2.66) with 40 yuan (approximately $6) for each one to engage in the experiment. Each participant needed to evaluate four entrepreneurial opportunities (i.e., two higher novel opportunities vs. two lower novel opportunities). For the validity of the evaluation materials, we conducted a pilot study and identified four opportunities with different degrees of novelty that were suitable for our research purposes before the formal experiment.

#### Opportunity Manipulation

Given that our research samples were a group of college students, and most of their entrepreneurial projects focused on technical products, we decided to use technical products as opportunity evaluation materials. After a discussion, we selected six entrepreneurial products from the entries in National Undergraduate Entrepreneurship Competition. Subsequently, we showed these products to 184 non-overlapping student participants who were reselected from multiple universities (58.7% female; *M*_age_ = 24.55 years; *SD* = 6.09) and requested them to score the novelty of each technical product. According to these scores, we chose two higher novel opportunities and two lower novel opportunities as rating targets. In our study, we created dummy variables for opportunity novelty as (1) and opportunity normal as (0).

Based on the above manipulation, we utilized the in-group design method to test the four hypotheses. Specifically, 80 Chinese college students were required to imagine that they were the CEOs of a high-tech start-up company within the laboratory environment. Subsequently, the participants were shown the same material, which contained the description and a sketch of each product, and were asked to score by themselves each technological entrepreneurship opportunity separately from two aspects of creativity perception and risk perception. After evaluation was complete, participants were asked to describe their thoughts during the evaluation of entrepreneurial opportunities. Meanwhile, they needed to record their opportunity adoption intention, the personal propensity of risk aversion, and openness through well-established scales. In addition, they also provided details of demographics. Similar to this process, each participant needed to complete the evaluation of four products (two higher novel opportunities vs. two lower novel opportunities).

### Measures

Our study measured dependent variable (i.e., opportunity adoption) and two mediators (i.e., creativity perception and risk perception) with well-established scales. We translated all English scales into Chinese using the double-back translation method (Brislin, 1980). Besides, we scored the moderator (i.e., construal level) using manual coding.

#### Dependent Variable: Opportunity Adoption

Using the three-item scale adapted from Lu et al. (2019), we asked all participants to score how they think about four products through three questions, including “if the opportunity is worthy of being further developed, transformed into a real product, and brought to the market.” The Cronbach's α was 0.93.

#### Mediators: Creativity Perception

We measured entrepreneurs' perceptions of creativity by using Lu et al.'s (2019) six-item scales. Each participant chose a rating ranging from *strongly disagree* (1) to *strongly agree* (7) on whether they perceive novelty and usefulness for each opportunity. The Cronbach's α was 0.86.

#### Risk Perception

We measured entrepreneurs' perceptions of risk by using Robert et al.'s (2009) six-item scales. Each participant scored each item as to how they perceived market risk or research and development risk for the opportunity with a seven-point Likert-type scale (1 = *strongly disagree*, 7 = *strongly agree*). The Cronbach's α was 0.85.

#### Moderator: Construal Level

We measured the participants' construal level based on three typical characteristics (i.e., abstraction, valence, and certainty) proposed by Magee et al. (2010). Specifically, after all participants completed the entrepreneurial opportunity evaluation, we asked each of them to recall the evaluation process and answer the following two questions: “What have you been thinking while evaluating the product?” and “Why would you consider these factors?” The researchers recorded the entire content of their answers. Next, aiming to ensure the objectivity of the measurement, two evaluators who did not know anything about the study were selected to code answers on a five-point scale to measure the three characteristics of construal level. We performed an interrater reliability test, and the results showed that ICC (3, 2) was 0.96 for abstraction, 0.94 for valence, and 0.93 for certainty based on the scoring scheme, which indicated high interrater agreement between two evaluators' scores. Ultimately, we averaged both the raters' scores on each of these three variables to measure the construal level.

#### Control Variables

Our study identified several key individual-level controls to exclude their interference. First, previous research has shown that openness in Big Five Personality Traits is most clearly linked with creativity (Feist, 1998; Hammond et al., 2011). This is because individuals with high openness usually have high imaginations for the same events and are more likely to engage in divergent thinking (McCrae, 1987), which may improve their creativity perception. Second, as a personality trait that determines willingness and propensity to take risks, the risk propensity of an individual is negatively related to his/her risk perception (Mandrik and Bao, 2005). Therefore, we included these two variables in the analysis to avoid their influence on creativity and risk perception by entrepreneurs. Additionally, we also collected the demographics of all participants such as gender, age, and education. However, we did not include them in our analysis because of fewer differences among all samples (Keh et al., 2002).

### Analytic Strategy

Accounting for the nested nature of the data in our study, we used a hierarchical linear model (HLM) to test our hypotheses. Construal level and two controls (i.e., the openness of Big-Five and risk propensity) were level 2 as well as other variables were level 1. Additionally, given that all the measures of the dependent variable (i.e., opportunity adoption) and two mediators (i.e., creativity perception and risk perception) were self-reports, the common method variance and social desirability bias might pose threats for the hypothesis testing. Therefore, we perform Harman's single-factor test to get exploratory factor analysis on all items. The results show that the number of factors exceeding one reached five. And the variance explained by the first main factor was 24.98%, which does not account for 40% of the total variation (66.97%). In other words, there is no serious common method variance and will not bring a substantial impact to our study.

## Results

Table 1 summarizes the descriptive statistics and correlations among all variables included in our model analyses. The results show that opportunity novelty has a positive relation with creativity perception (*r* = 0.51, *p* < 0.01) and risk perception (*r* = 0.38, *p* < 0.01), respectively. Meanwhile, there is a positive relation between creativity perception and opportunity adoption (*r* = 0.46, *p* < 0.01) but a negative relation between risk perception and opportunity adoption (*r* = −0.20, *p* < 0.01). All of them provide the preliminary support for hypothesis testing.

**Table 1:** Descriptive statistics and correlations among all variables.

**Variable**	***M***	***SD***	**1**	**2**	**3**	**4**
**PRODUCT LEVEL**						
**Level 1 variables**						
1. Opportunity novelty	0.50	0.50	1.00			
2. Creativity perception	4.39	1.27	0.51^**^	1.00		
3. Risk perception	3.09	1.16	0.38^**^	0.13^*^	1.00	
4. Opportunity adoption	5.03	1.37	0.05	0.46^**^	−0.20^**^	1.00
**Level 2 variables**						
1. Risk propensity	4.55	0.74	1.00			
2. Openness of Big-Five	3.58	0.77	−0.30^**^	1.00		
3. Construal level	3.54	0.47	−0.02	0.20^**^	1.00	

*N (Level 1) = 320; N (Level 2) = 80;*

** *p < 0.01;*

* *p < 0.05*.

We conducted HLM analyses to test the first hypothesis. Opportunity novelty was entered as the predictor at Level 1. To examine the moderating effect of construal level on the relation between opportunity novelty and creativity perception, we tested the interaction of construal level and opportunity novelty in the Level 1 model. Specifically, we first estimated a null model to examine the between-rater variability of the intercept and intraclass correlation coefficient (ICC1) for the dependent variable, creativity perception. The result showed that there was significant between-rater variance for creativity perception (intercept = 0.087, *p* < 0.05, ICC1 = 0.105). And after entering opportunity novelty in the model, we found that the random intercept and the random slope were significant (intercept = 0.265, *p* < 0.01, slope variance = 0.273, *p* < 0.1). Therefore, it was appropriate to use HLM to analyze our data and the results were displayed in Table 2. For creativity perception, the interaction between opportunity novelty and construal level was non-significant (γ = 0.264, *p* > 0.1). Hence, Hypothesis 1 was not supported.

**Table 2:** HLM results for the interactive effect of opportunity novelty and construal level on creativity perception.

	**(1) Creativity perception**	**(2) Creativity perception**	**(3) Creativity perception**	**(4) Creativity perception**
**Level 1 variables**				
Intercept	4.778^***^ (0.058)	4.527^***^ (0.083)	3.589^***^ (0.655)	4.144^***^ (0.800)
Opportunity novelty		0.500^***^ (0.103)	0.500^***^ (0.103)	−0.432 (0.777)
**Level 2 variables**				
Risk propensity			0.045 (0.082)	0.045 (0.082)
Openness of Big Five			0.026 (0.081)	0.026 (0.081)
Construal level			0.182 (0.126)	0.025 (0.181)
**Cross-level interaction**				
Opportunity novelty × Construal level				0.264 (0.218)
Residual variance	0.740	0.569	0.569	0.569
Intercept	0.087	0.265	0.281	0.280
Slope variance		0.273	0.273	0.268
AIC	851.405	825.850	838.036	839.782
BIC	862.710	848.460	871.951	877.466
Deviance	845.405	951.808	946.019	941.037

*Unstandardized regression coefficients are displayed, with standard errors in parentheses*.

*N (Level 1) = 320; N (Level 2) = 80;*

*** *p < 0.001*.

**Figure 2:**
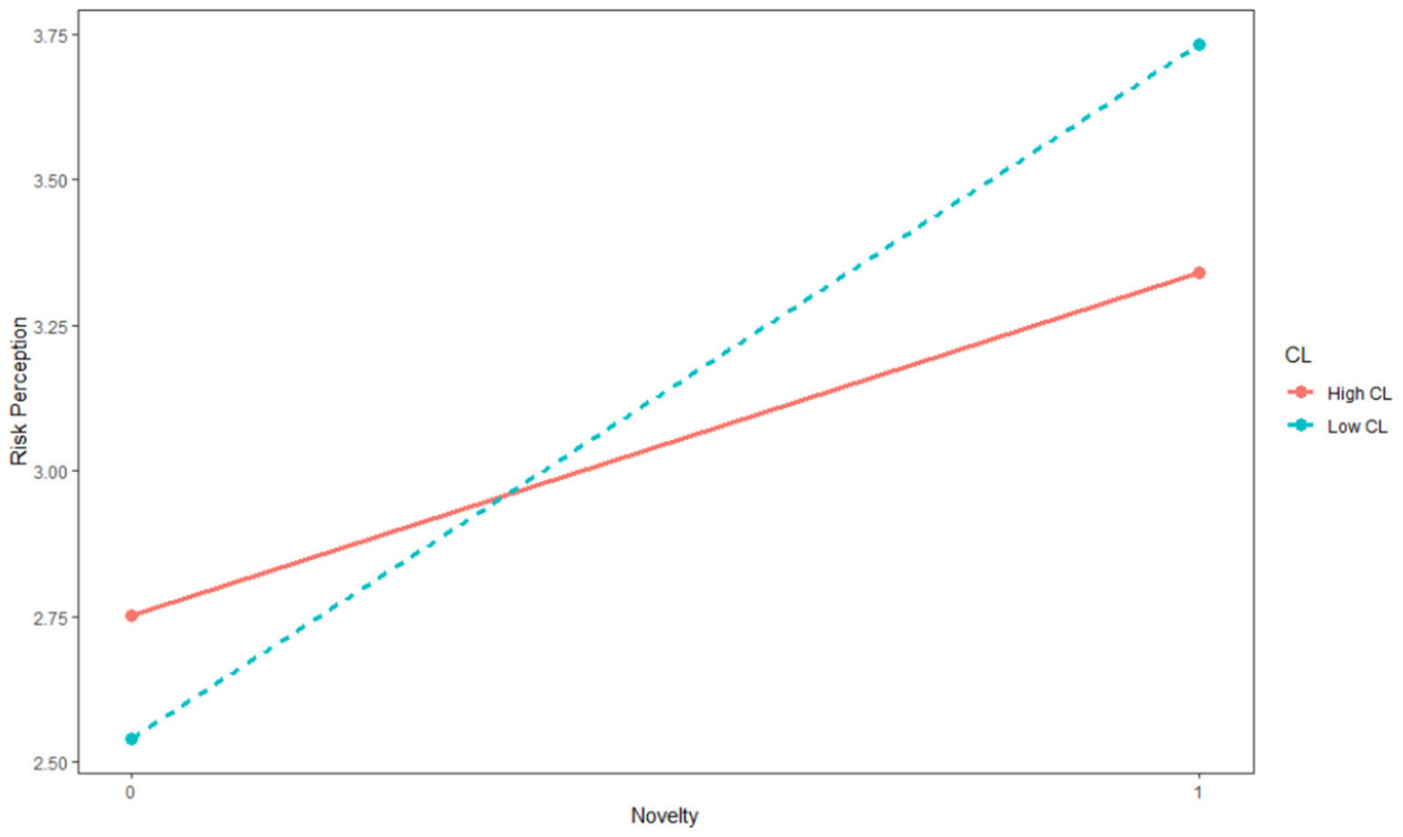
The interactive effect of opportunity novelty and construal level on risk perception.

Likewise, we also ran HLM and the result of the second hypothesis was displayed in Table 3. We found that the interaction between opportunity novelty and construal level was negatively significant for risk perception (γ = −0.642, *p* < 0.05). To better demonstrate the interactive effect of construal level and opportunity novelty on risk perception, we conducted a simple slopes test as illustrated in Figure 2. The results revealed that the relationship between opportunity novelty and risk perception was more positively significant when entrepreneurs' construal level was low, whereas the positive effect was weaker when their construal level was high. Hence, Hypothesis 2 was supported.

**Table 3:** HLM results for the interactive effect of opportunity novelty and construal level on risk perception.

	**(1) Risk perception**	**(2) Risk perception**	**(3) Risk perception**	**(4) Risk perception**
**Level 1 variables**				
Intercept	3.091^***^ (0.065)	2.646^***^ (0.098)	2.017^**^ (0.666)	0.700 (0.853)
Opportunity novelty		0.891^***^ (0.127)	0.891^***^ (0.127)	3.161^***^ (0.940)
**Level 2 variables**				
Risk propensity			0.279^***^ (0.084)	0.281^***^ (0.084)
Openness of Big Five			−0.036 (0.082)	−0.035 (0.082)
Construal level			−0.145 (0.128)	0.224 (0.197)
**Cross-level interaction**				
Opportunity novelty × construal level				−0.642^*^ (0.264)
Residual variance	1.346	1.020	1.000	0.995
Intercept	0.000	0.262	0.203	0.179
Slope variance		0.279	0.287	0.230
AIC	1011.914	963.808	964.019	961.037
BIC	1023.219	986.418	997.934	998.720
Deviance	1005.914	951.808	946.019	941.037

*Unstandardized regression coefficients are displayed, with standard errors in parentheses*.

*N (Level 1) = 320; N (Level 2) = 80;*

*** *p < 0.001;*

** *p < 0.01;*

* *p < 0.05*.

Subsequently, to test the third and fourth hypothesis, we conducted HLM again to examine the indirect effect in moderated mediation. The Level 1 predictor (opportunity novelty) was group-mean centered and the Level 2 variable (construal level) was grand-mean centered (Aguinis et al., 2013). The results (Tables 4, 5) indicated that the indirect effect of opportunity novelty on opportunity adoption via creativity perception was, respectively, significant when the construal level of entrepreneurs was high (γ = 0.738, 95%CI = [0.393, 1.082]) and low (γ = 0.445, 95%CI = [0.092, 0.798]). However, the difference between these indirect effects was not significant (Δ γ = 0.293, 95%CI = [−0.197, 0.782]). In other words, creativity perception of entrepreneurs mediated the relationship between opportunity novelty and opportunity adoption. But the moderated mediation effect on the relation between opportunity novelty and opportunity adoption was not significant. Hence, Hypothesis 3 was not supported.

**Table 4:** Results for the mediating effect of creativity perception on the relation between opportunity novelty and opportunity adoption.

	**(1)**	**(2)**	**(3)**
	**Opportunity adoption**	**Creativity perception**	**Opportunity adoption**
(Intercept)	4.518^***^ (0.719)	4.162^***^ (0.457)	−0.374 (0.536)
Opportunity novelty	0.132 (0.154)	0.500^***^ (0.098)	−0.457^***^ (0.106)
Risk propensity	0.016 (0.109)	0.037 (0.070)	−0.027 (0.073)
Openness of Big Five	0.105 (0.106)	0.055 (0.067)	0.040 (0.070)
Creativity perception			1.175^***^ (0.059)
*R* ^2^	0.005	0.078	0.562
Adj. *R*^2^	−0.004	0.070	0.557

*Unstandardized regression coefficients are displayed, with standard errors in parentheses*.

*N = 320;*

*** *p < 0.001*.

**Table 5:** Results for the moderated mediation.

	**Path**	**Moderator**	***r***	**95% CI**
Indirect	Opportunity novelty—creativity	High construal level	0.738^***^	[0.393, 1.082]
effect	perception—opportunity adoption	Low construal level	0.445^*^	[0.092, 0.798]
		Difference	0.293	[−0.197, 0.782]

*N (Level 1) = 320; N (Level 2) = 80;*

*** *p < 0.001;*

* *p < 0.05*.

For the fourth hypothesis, the results (Tables 6, 7) showed that the indirect effect of opportunity novelty on opportunity adoption via risk perception was negatively significant when the construal level of entrepreneurs was high (γ = −0.202, 95% CI = [−0.353, −0.052]) and low (γ = −0.410, 95% CI = [−0.643, −0.176]). In addition, the difference between these indirect effects was also significant (Δ γ = 0.207, 95% CI = [0.003, 0.411]). Hence, Hypothesis 4 was supported.

**Table 6:** Results for the mediating effect of risk perception on the relation between opportunity novelty and opportunity adoption.

	**(1)**	**(2)**	**(3)**
	**Opportunity adoption**	**Risk perception**	**Opportunity adoption**
(Intercept)	4.518^***^ (0.719)	1.418^*^ (0.548)	4.967^***^ (0.706)
Opportunity novelty	0.132 (0.154)	0.891^***^ (0.117)	0.414^*^ (0.163)
Risk propensity	0.016 (0.109)	0.303^***^ (0.083)	0.112 (0.109)
Openness of Big Five	0.105 (0.106)	−0.042 (0.081)	0.092 (0.103)
Risk perception			−0.317^***^ (0.072)
*R* ^2^	0.005	0.189	0.063
Adj. *R*^2^	−0.004	0.181	0.052

*Unstandardized regression coefficients are displayed, with standard errors in parentheses*.

*N = 320;*

*** *p < 0.001;*

* *p < 0.05*.

**Table 7:** Results for the moderated mediation.

	**Path**	**Moderator**	***r***	**95% CI**
Indirect	Opportunity novelty—risk	High construal level	−0.202^**^	[−0.353, −0.052]
effect	perception—opportunity adoption	Low construal level	−0.410^***^	[−0.643, −0.176]
		Difference	0.207^*^	[0.003, 0.411]

*N (Level 1) = 320; N (Level 2) = 80;*

*** *p < 0.001;*

** *p < 0.01;*

* *p < 0.05*.

## Discussion

Based on information processing theory and CLT, this paper constructs and examines the cognitive evaluation process from opportunity novelty to opportunity adoption. Through a situational experiment, we discover that opportunity novelty will interact with construal level to shape entrepreneurs' perceptions, which in turn affect decisions of opportunity adoption. Specifically, entrepreneurs using a low-level construal perceive higher risk for the opportunity novelty, which in turn decreases the probability of opportunity adoption. Meanwhile, we also find that opportunity novelty is positively related to creativity perception of entrepreneurs, which in turn increases the possibility of adopting this opportunity. However, it is not proven that there is an interactive effect of opportunity novelty and construal level between on creativity perception of entrepreneurs, which may be caused for several reasons as follows.

First, similar to the common practice in prior studies, creativity perception was measured in our study through two aspects of novelty and usefulness that are usually combined in a single construct (Mueller et al., 2018; Du et al., 2021). However, some evidence suggests that dimensions of novelty and usefulness are orthogonal (Litchfield, 2008), and perceptions of them are conflicting (e.g., Mueller et al., 2012; Berg, 2016) as they are motivated by opposing evaluative processes such that the more novel an opportunity, the more uncertainty exists about its usefulness (Miron-Spektor et al., 2011). High-level construal will increase entrepreneurs' novelty perception and decrease their usefulness perception (Mueller et al., 2014). As such, the effect of construal level on perceptions of novelty and usefulness may be offset by each other, which leads to the result that the moderating effect of construal level on the relation between opportunity novelty and creativity perception is insignificant. Meanwhile, the fact has been found by Sonenshein (2014) in his recent study that usefulness perception of any product is dynamic and complex as well as easily manipulated and changed, which suggests that usefulness perception for a novel opportunity may also be affected by other factors apart from construal level. Ultimately, there is no significance in the interactive effect of opportunity novelty and construal level on entrepreneurs' creativity perception due to the joint effect of other factors. Future research therefore can take time to investigate differences in the effect of construal level and other factors on two components of creativity perception—perceptions of novelty and usefulness—in the process of opportunity novelty evaluation.

### Theoretical Contributions

Our study makes theoretical contributions to the existing research in several ways. First, this paper, building on information processing theory, contributes to the literature on opportunity evaluation by providing empirical evidence for the cognitive information processing process from opportunity novelty to opportunity adoption, which helps explain why some entrepreneurs reach different adoption decisions for the same opportunity novelty. Accurate evaluation of opportunity novelty is critical to entrepreneurial activity, which determines whether an individual adopts the novel opportunity to start a new business or the internal entrepreneurship in an established firm (Nicolau et al., 2009). In this study, we clarify the cognitive evaluation mechanisms between opportunity novelty and opportunity adoption based on information processing theory such that individuals' subjective perceptions of objects will influence their behavioral decisions. Our results indicate that the objective characteristic of opportunity, instead of directly affecting entrepreneurial opportunity adoption, interacts with construal level to shape entrepreneurs' perceptions in terms of gains and losses, which, in turn, influence their adoption decisions. Generally speaking, our findings directly responds to the longstanding call that “the basic process of opportunity evaluation has still been ignored” (Haynie et al., 2009) by further explaining why people have different perceptions of opportunity attraction and how they make decisions to adopt or reject novelty opportunities.

Second, our study further enriches the application contexts of CLT by introducing it combined with information processing theory to the field of entrepreneurship and identifying individuals' construal level as a critical factor in shaping the cognitive evaluation process of opportunity novelty. Opportunity evaluation is essentially a process of judgment based on cognition (Shepherd et al., 2007; Wood and McKelvie, 2015). According to information processing theory, opportunity evaluation is strongly affected by how entrepreneurs interpret information (Hambrick, 2007). Construal level theory includes the belief that individuals' construal levels can expand and contract their mental horizon by representing objects ranging from abstract to concrete (Trope and Liberman, 2003), which, in turn, affects their final judgments and decisions (Liberman et al., 2007; Trope and Liberman, 2010). Thus, positioning construal level as an essential information representation device in the process of opportunity novelty evaluation, we demonstrate that different levels of construal can shape the perceptions of entrepreneurs interacting with opportunity novelty, which in turn increases or decreases the probability of opportunity adoption. In summary, our results emphasize the usefulness of applying CLT to the opportunity evaluation process and may provide new perspectives for intervention techniques during opportunity novelty evaluation.

Third, it is worth noting that our paper, focusing on the novelty characteristic of opportunity, augments the literature on outcomes of novelty by analyzing the impact of novelty on adoption decisions. Existing entrepreneurial research mainly emphasizes the discovery, utilization, and results of opportunities and is less concerned with the nature of opportunities (McMullen et al., 2007), which hinders the development of opportunity theory to a certain extent. In fact, it is crucial to pay more attention to the characteristics of opportunity to understand the nature of opportunity evaluation (Mount et al., 2021), especially for opportunity novelty. Because as a typical characteristic of entrepreneurial opportunities, diverse degrees of opportunity novelty have differentiated value creating potential (Aldrich and Martinez, 2001), which is essential to the development of start-ups. As such, focusing on novelty characteristic, we add to the literature on outcomes of novelty by further clarifying how opportunity novelty affects opportunity adoption decisions.

### Practical Implications

There are also some implications for entrepreneurship practice and education. First of all, entrepreneurs and investors can better understand the cognitive evaluation process from opportunity novelty to opportunity adoption through our study, which helps them effectively avoid cognitive biases when evaluating opportunity novelty. Specifically, people need to evaluate novel opportunities first to determine whether they are worthy of further action (Haynie et al., 2009; Wood and McKinley, 2010). Our results indicate that individuals' construal levels can, interacting with opportunity novelty, shape their perceptions of opportunity, which in turn leads to different judgments and decisions. Therefore, entrepreneurs and investors should be aware of their construal levels and consciously adjust them when evaluating opportunity novelty. For example, they should use a high-level construal to reduce their perceptions of risk and then increase the possibility of adopting novel opportunities in a business environment where novelty actions have to occur.

Second, firms can choose partners with different construal levels to develop their businesses according to our study. Specifically, the decision of whether to adopt a novel opportunity is usually made by the whole entrepreneurial team even though that entrepreneurs play a central role in entrepreneurial activities (West, 2007). The results underscore that even for opportunities with the same level of novelty, individuals using different levels of construal may generate multi-degrees of perceptions. An individual using a high-level construal perceives fewer perceptions of risk and is more likely to adopt the novel opportunity. That is to say, using a high-level construal can help entrepreneurs make decisions of opportunity adoption for high opportunity novelty. Thus, when faced with the challenge of developing or exploiting novel opportunities, firms should focus on choosing entrepreneurial partners or senior management members with high-level construals in a targeted manner to increase favorable decisions to adopt highly novel opportunities.

Third, entrepreneurial educators should consider further supplementing the cognitive management course of opportunity evaluation, which aims to enable potential entrepreneurs among students to understand the role of their subjective cognition in the process of evaluating characteristics of opportunities. Entrepreneurship plays a vital role in innovation vitality and economic growth for countries and regions (Ravenelle, 2019). Therefore, many government programs and University courses view entrepreneurship education as an important means of cultivating students' entrepreneurial spirit and stimulating their entrepreneurial potential (Tumasjan et al., 2013). Most entrepreneurial courses focus on entrepreneurial skills such as how to develop business plans and identify opportunities (Foo, 2011). In the future, courses about opportunity evaluation and proactive intervention techniques in entrepreneurship education should be included to help potential entrepreneurs make more accurate evaluations of novel opportunities, which is conducive to the establishment and development of new ventures.

### Limitations and Future Research

A few limitations are worth noting in interpreting and citing the results of our study. First, our paper provided convincing empirical evidence for the cognitive evaluation process from opportunity novelty to opportunity adoption based on information processing theory and CLT. However, entrepreneurial opportunities also have other characteristics such as practicality and uniqueness (Baron and Markman, 2003). Entrepreneurs may adopt different cognitive processes when evaluating opportunities with different characteristics. Future research should explore the cognitive process of entrepreneurial opportunity evaluation from other opportunity characteristics to form a more comprehensive understanding of opportunity evaluation. Second, our research focuses on the individual level to explore the cognitive evaluation process of entrepreneurs to adopt novel opportunities. Indeed, such opportunity adoption decisions are sometimes made by collectives (Mount et al., 2021). Some scholars argue that groups may adopt different criteria when new opportunities are evaluated in different modes such as a sequential or parallel mode (Harvey and Kou, 2013). Therefore, future research should further examine how entrepreneurial teams make adoption decisions for novel opportunities and compare differences in the opportunity evaluation process between individual and group levels. Third, our research may include the challenge of external validity because we conducted an experiment instead of observing actual entrepreneurial behavior. The experimental settings do not fully reflect a natural decision-making environment (Aguinis and Bradley, 2014; Gruber et al., 2015). In our study, participants evaluated novel opportunities by imagining they were the CEOs of a high-tech start-up firm in a laboratory rather than in the actual business environment, which may have simplified the decision-making environment to a certain extent. We recommend that future research explore the cognitive process of an individual's opportunity evaluation in a natural environment.

## Data Availability Statement

The data sets generated for this study are available upon request to the corresponding author.

## Ethics Statement

The studies involving human participants were reviewed and approved by Academic Committee of business school, Central University of Finance and Economics. The patients/participants provided their written informed consent to participate in this study.

## Author Contributions

All authors listed have made a substantial, direct and intellectual contribution to the work, and approved it for publication.

## Funding

This research was supported by the National Natural Science Foundation of China (71971227; 72072192).

## Conflict of Interest

The authors declare that the research was conducted in the absence of any commercial or financial relationships that could be construed as a potential conflict of interest.

## Publisher's Note

All claims expressed in this article are solely those of the authors and do not necessarily represent those of their affiliated organizations, or those of the publisher, the editors and the reviewers. Any product that may be evaluated in this article, or claim that may be made by its manufacturer, is not guaranteed or endorsed by the publisher.
